# Bioinformatics analysis of the KEA gene family in rice

**DOI:** 10.1515/biol-2025-1230

**Published:** 2026-03-18

**Authors:** Zhenyu Zhang, Shu Dang

**Affiliations:** Heilongjiang Bayi Agricultural University, Daqing, 163000, China; Jilin Agricultural Science and Technology University, Jilin, 132101, China

**Keywords:** bioinformatics analysis, KEA gene family, rice, tissue expression

## Abstract

KEA (K^+^/H^+^ reverse transporter, K^+^ efflux antiporter) gene family belongs to the CPA2 reverse transporter superfamily gene, which plays an important role in regulating plant growth and development and stress resistance by maintaining intracellular PH balance and K^+^ concentration. The studies on the function of KEA gene in plants mainly focus on *Arabidopsis thaliana*, and the studies on the function of KEA gene family in rice have not been reported. In this study, the evolutionary tree analysis, chromosome localization, protein physicochemical properties and tissue expression of rice KEA gene family were analyzed by bioinformatics methods. Eight KEA family genes were identified in rice, some localized on specific chromosomes, with particular domains and different expression patterns. These results provided references for the research and analysis of the function of rice KEA gene family.

## 1 Introduction

Rice (*Oryza sativa* L.) is presently the world’s most important cereal grain, with one-fourth of the population depending on it as a major stable food [1], 2]. Rice constitutes approximately 40 % of China’s total annual grain production. Its sowing area and annual output rank first among all grain crops. Revered as the leader of the five grains, rice has served as a staple food for the Chinese people for 4,000 years [3].

Potassium ions (K^+^) are the most abundant cations in plant cells, regulating plant growth and development as well as stress response through various physiological processes such as transmembrane transport, enzyme activation, anion neutralization, photosynthesis, osmotic regulation, stomatal movement, etc. [4], [5], [6]. The growth and development of plants require the absorption and transport of K^+^ ions, and in these processes, both K^+^ channel proteins and K^+^ transporters are indispensable [7]. The cooperation of these two proteins can promote the absorption and transport of K^+^ in plants. K^+^ transporters not only regulate the concentration of K^+^ in plant cells and maintain the balance of pH in plant cells, but also play a very important role in plant stress resistance and pollen growth and development [8], 9].

Some researchers have reported the mechanistic effects of K^+^ on plant disease resistance. The higher K^+^ concentration reduces the internal competition of pathogens for nutritional resources [10], 11]. This nutritional state enables plants to allocate more resources to develop stronger cell walls, prevent pathogen infections and insect attacks, and obtain more nutrients for plant defense and damage repair [12]. During airborne pathogen infections (especially bacterial and viral infections), when there is sufficient K^+^, stomata can function normally, preventing pathogen invasion by quickly closing stomata. K^+^ is also essential for the function of various plant enzymes, regulating the metabolic patterns of plants and ultimately changing metabolite concentrations. In plants with sufficient K^+^, the synthesis of compounds (such as proteins, starch, and cellulose) significantly increases, thereby reducing the concentration of low molecular weight compounds (such as soluble carbohydrate organic acids, amino acids) and amides in plant tissues [13]. These low molecular weight compounds are important for the development of infections and pests, therefore lower concentrations make plants less susceptible to disease and pest invasion in K^+^- rich plants [11]. The sufficient potassium can increase the concentration of phenols, which plays a key role in plant resistance [12]. In addition, the reduced pest damage in high potassium plants can be attributed to the lack of pest preference at sufficient nutrient concentrations, leading to higher pest mortality rates due to the synthesis of defensive compounds [13].

K^+^/H^+^ antiporters are secondary transporters found in various organisms, including bacteria, yeast, plants, and animals. They are H^+^ coupled co transporters, and their biochemical activity involves transferring K^+^ through membranes to exchange protons [14]. K^+^/H^+^ antiporters form a large gene family, with over 200 genes annotated as K^+^/H^+^ antiporters. Cation proton antiporters (CPAs) can mediate cation efflux and H^+^ influx in plant cells [15]. They are K^+^/H^+^ antiporters widely present in plants, animals, bacteria, and fungi, mainly located on the vacuolar membrane and cytoplasmic membrane, as well as on the organelle membrane in plants [16], 17]. Plant cation proton antiporter CPAs are divided into two subfamilies: CPA1 and CPA2. The CPA1 subfamily contains NHX (Na^+^/H^+^ exchanger) transporter genes, while the CPA2 subfamily contains KEA (K^+^/H^+^ antiporter) and CHX (Cation/H^+^ exchanger) transporter genes [18]. In the *Arabidopsis* genome, approximately 44 genes are predicted to encode K^+^/H^+^ antiporters, including 8 AtNHXs, 28 AtCHXs, and 6 AtKEAs [19]. In recent decades, research on plant K^+^/H^+^ antiporters has mainly focused on NHX and CHX genes, and research on the KEA gene family is rare. The KEA gene is involved in plant growth and development, as well as many physiological and biochemical processes of cells, regulating external stress [20]. Studies have shown that the KEA gene is most expressed in pre flowering and flowering pollen, suggesting that the KEA gene can effectively promote flower development and opening [21], 22]. Until today, there have been many studies on the absorption, transport, unloading, and distribution of K^+^, mainly in *Arabidopsis*. Six KEA reverse transport genes were first identified in *Arabidopsis* [18], 19]. Although the KEA family has been functionally characterized in model plants such as *Arabidopsis*, research on KEA genes in crops remains limited. For example, Rehman et al. [23] identified 12 GmKEA genes in soybean and demonstrated that most are transcriptionally involved in responses to abiotic stresses such as potassium deficiency and salt stress. Sharma et al. [24] analyzed the expression profiles of 24 TaKEA genes in wheat and proposed their potential roles in growth and stress adaptation. In maize, Kong et al. [25] identified six ZmKEA members and revealed the salt tolerance function of ZmKEA2 through functional complementation in yeast and analysis of zmkea2 mutants. In rice, although three OsKEA members have been reported, e.g., OsKEA1, which functions as a K^+^/H^+^ antiporter regulating the proton gradient across thylakoid membranes [22], a comprehensive genome-wide identification and functional analysis of all OsKEA genes is still lacking. Therefore, systematic identification and characterization of the OsKEA family will help elucidate the mechanisms underlying K^+^ uptake and salt stress tolerance in rice.

Saline-alkali stress is a major constraint on global rice production. Developing saline-alkali tolerant rice varieties has thus become a key focus in rice breeding. However, the plant’s response to saline-alkali stress involves a complex regulatory network comprising multiple signaling pathways and is influenced by various factors [26]. As a result, the molecular mechanisms underlying rice tolerance remain insufficiently studied. Additionally, rice cultivation faces significant K^+^ deficiency, particularly in southern China. The KEA family genes are known to play important roles in K^+^ uptake and salt stress resistance in plants. Given the functional and expressional diversity of KEA members in *Arabidopsis*, investigating the KEA gene family in rice will provide a critical foundation for elucidating its roles in this major crop. This article provides technical support for the future development of rice breeding and industry by studying the composition and expression patterns of the KEA gene family in rice.

## 2 Materials and methods

### 2.1 Identification of rice KEA gene family

First, the complete genome data and annotation files of rice were downloaded from Ensembl Plants (release 62), and KEA protein sequences of *Arabidopsis thaliana* were obtained from Plant Genomic Information Resources (http://plantgir.cn/). Using the “Blast Several Sequences to Big Database” function in TBtools-II software, a BLAST analysis was performed against the entire rice protein dataset with the *Arabidopsis* KEA protein sequences as queries, applying an E-value cutoff of 1 × 10^−10^. The initial matches were subsequently subjected to conserved domain validation via the Batch CD-search tool on the NCBI website (https://www.ncbi.nlm.nih.gov/Structure/bwrpsb/bwrpsb.cgi). Only sequences containing the Na_H_Exchanger domain with an E-value less than 0.01 were retained.

### 2.2 Conserved domain analysis of amino acid sequence of rice KEA gene family protein

By using the online program CDD in NCBI (https://www.ncbi.nlm.nih.gov/) to predict the conserved domain, genes with specific protein domains were selected as the target sequences of rice KEA gene family proteins needed in this study and Tbtools software was used for mapping.

### 2.3 Mapping of rice KEA gene on chromosome

The position of rice KEA gene on chromosome was queried in Ensembl Plants database (release 62), and then TBtools software was used for visualization processing.

### 2.4 Analysis of physicochemical properties of rice KEA gene protein

The physicochemical properties, including molecular weight and isoelectric point, of the rice KEA gene family members were predicted based on their amino acid sequences using the Compute pI/Mw tool available on the Expasy online server.

### 2.5 Subcellular localization of rice KEA gene protein

Based on the amino acid sequences of the KEA gene family members in rice, subcellular localization prediction analysis was conducted for this gene family using the Plant-PLoc online tool (http://www.csbio.sjtu.edu.cn/bioinf/plant-multi/).

### 2.6 KEA gene family tree analysis in rice and *A. thaliana*

To construct the phylogenetic tree, multiple sequence alignment of KEA family protein sequences from rice and *A. thaliana* was performed using the MUSCLE tool implemented in MEGA11. A phylogenetic tree was then generated using the Neighbor-Joining method with the following parameters: 1,000 bootstrap replicates, Poisson model, and pairwise deletion.

### 2.7 Analysis of gene structure and motif of rice KEA gene family

The gene structure of 8 KEA genes in rice was visualized by Tbtools software. motif prediction was carried out using MEME online tool (https://meme-suite.org/meme/tools/meme), and the number of motifs was set to 10. Other conditions remained unchanged with the system, and the existing OsKEA protein sequences were predicted.

### 2.8 Tissue expression of rice KEA gene family

The FPKM value of the gene family in each tissue was obtained in the Rice Expression Database (IC4R-2.0), and then Tbtools software was used for visualization processing, and finally the heat map of the expression of different tissues of the rice KEA gene family was searched.

## 3 Results

### 3.1 Identification of rice KEA gene family

Based on 6 *Arabidopsis* KEA genes annotated in *Arabidopsis* Tair database, a total of 8 KEA genes were found in rice by Blast sequence similarity analysis combined with conserved domain prediction results. Locus numbers in the database are LOC_Os01g60140, LOC_Os03g03590, LOC_Os04g58620, LOC_Os05g40650, LOC_Os05g19500, LOC_Os06g36590, LOC_Os12g42300, LOC_Os12g42200, respectively. In this study, the above eight genes were named OsKEA1, OsKEA2, OsKEA3, OsKEA4, OsKEA5, OsKEA6, OsKEA7, OsKEA8.

### 3.2 Analysis of rice KEA gene domain

**Figure 1:**
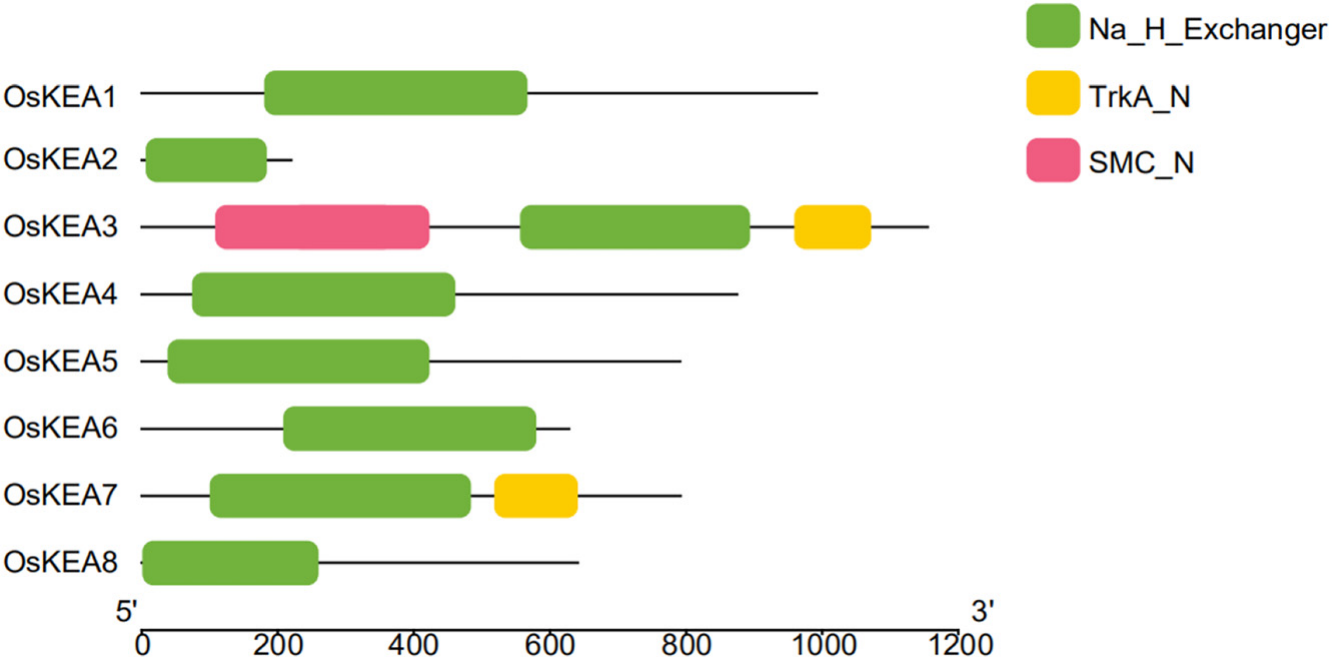
Conservative domain analysis of rice KEA gene family. Notes: Different domains are shown by a rectangle with different colors. The scale represents the length of the protein, and all proteins are displayed in proportion.

The KEA family proteins in rice exhibits similarities to its counterpart in *Arabidopsis*. The rice KEA proteins contain three distinct domains: Na_H_Exchanger, TrkA_N, and SMC_N. Our analysis revealed that the Na_H_Exchanger conserved domain is present in all OsKEA proteins and represents the primary functional region (Figure 1). In addition to the Na_H_Exchanger domain, OsKEA3 possesses two additional domains: TrkA_N and SMC_N. Proteins belonging to the structural maintenance of chromosomes (SMC) superfamily typically feature ATP-binding domains at their N-termini. Accordingly, the SMC_N domain is hypothesized to be involved in chromatin organization and DNA dynamics, suggesting that OsKEA3 may have acquired a novel function.

### 3.3 Localization of rice KEA gene on chromosomes

**Figure 2:**
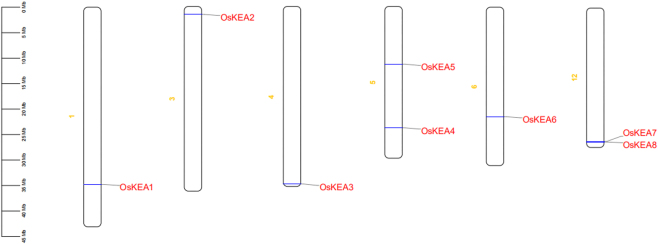
Position of KEA gene on different chromosomes of rice. Notes: Different chromosomes are labeled on the left. The scale indicates the length of the chromosomes, and all chromosomes are presented in proportion.

By comparing the relevant information of the rice KEA genome, the distribution of eight members of the rice KEA gene family on the 12 rice chromosomes was obtained (Figure 2). We found that the first, third, fourth, and sixth chromosomes are respectively distributed with OsKEA1, OsKEA2, OsKEA3, and OsKEA6 genes, all located at one end of the chromosome; On the fifth chromosome, OsKEA4 and OsKEA5 genes are distributed separately, while on the twelfth chromosome, OsKEA7 and OsKEA8 genes are distributed, and the two KEA genes are arranged in a gene cluster. This dispersed distribution reflects an ancient evolutionary origin of the KEA family in rice, while the clustering of OsKEA7 and OsKEA8 suggests a recent gene duplication event.

### 3.4 Analysis of physicochemical properties of KEA gene protein in rice

The KEA gene family in rice is shown in Table 1. Overall, the number of amino acids ranges from 219 to 1,140; The molecular weight ranges from 122,422.48 to 24,336.18; The fat coefficient ranges from 102.40 to 133.52. The average isoelectric point is about 6.7. The average hydrophilicity coefficients are all positive, indicating poor fluidity. The fat coefficient value is between 102.40 and 133.52. Overall, the KEA gene family in rice has strong protein stability.

**Table 1:** Analysis of physical and chemical properties of KEA gene family proteins.

Name	Amino acid length (aa)	Molecular weight (Da)	PI	Average of hydropathicity	Aliphatic index
OsKEA1	991	107,589.91	7.25	0.269	102.40
OsKEA2	219	24,336.18	7.85	0.912	133.52
OsKEA3	1,140	122,422.48	5.13	0.091	104.36
OsKEA4	874	93,260.23	6.69	0.503	111.68
OsKEA5	790	84,937.39	7.02	0.410	112.33
OsKEA6	627	66,212.87	5.69	0.681	124.70
OsKEA7	791	85,343.24	5.49	0.327	114.54
OsKEA8	640	68,584.05	8.49	0.330	105.50

### 3.5 Subcellular localization of KEA gene proteins in rice

The subcellular localization of rice KEA family proteins was predicted. As summarized in Table 2, OsKEA1, OsKEA2, OsKEA4, OsKEA5, OsKEA6, and OsKEA7 are all localized to the vacuolar membrane. Among these, OsKEA2, OsKEA4, OsKEA5, and OsKEA6 are simultaneously targeted to both the plasma membrane and the vacuolar membrane. In contrast, OsKEA1, OsKEA7, and OsKEA8 are exclusively localized to the vacuolar membrane, while OsKEA3 is uniquely predicted to reside in the nucleus. The predominant localization of most members to the plasma and vacuolar membranes supports the hypothesis that this protein family may function as transport proteins, potentially serving as cation/proton antiporters in rice. This characteristic further implies that the OsKEA family may play a critical role in maintaining intracellular K^+^ homeostasis and osmotic regulation.

**Table 2:** Subcellular location prediction of OsKEAs.

Name	LOCUS	Subcellular location prediction
OsKEA1	LOC_Os01g60140	Vacuole
OsKEA2	LOC_Os03g03590	Plasma membrane, Vacuole
OsKEA3	LOC_Os04g58620	Nucleus
OsKEA4	LOC_Os05g40650	Plasma membrane, Vacuole
OsKEA5	LOC_Os05g19500	Plasma membrane, Vacuole
OsKEA6	LOC_Os06g36590	Plasma membrane, Vacuole
OsKEA7	LOC_Os12g42300	Vacuole
OsKEA8	LOC_Os12g42200	Vacuole

### 3.6 Phylogenetic tree analysis of rice KEA gene family in rice and *A. thaliana*

**Figure 3:**
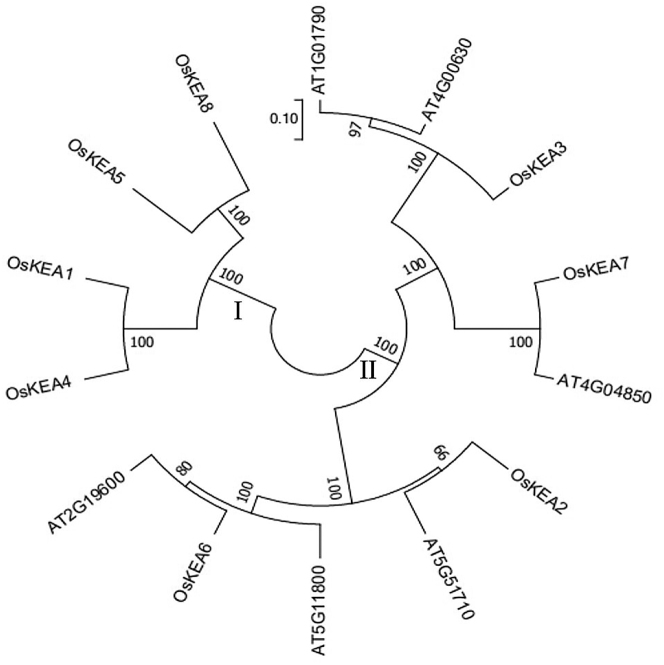
Evolutionary tree of rice KEA gene family.

Compare the identified eight rice KEA gene sequences and 6 *Arabidopsis* KEA gene sequences and construct a phylogenetic tree. According to Figure 3, the KEAs members of the two crops mentioned above are divided into two subgroups, named Group I and Group II, respectively. In Figure 3, it can be seen that the self-expansion value is generally 100, indicating high reliability. There are four genes in Group I (OsKEA1, OsKEA4, OsKEA5, OsKEA8) that are all rice genes, indicating that these four rice genes have a relatively distant evolutionary relationship with genes in *Arabidopsis*; Group II consists of 10 genes, all of which are four rice varieties (OsKEA2, OsKEA3, OsKEA6, OsKEA7) and six *A. thaliana* members. These four rice genes are closely related to the genes in *Arabidopsis*.

### 3.7 Gene structure and motif analysis of rice KEA gene family

**Figure 4:**
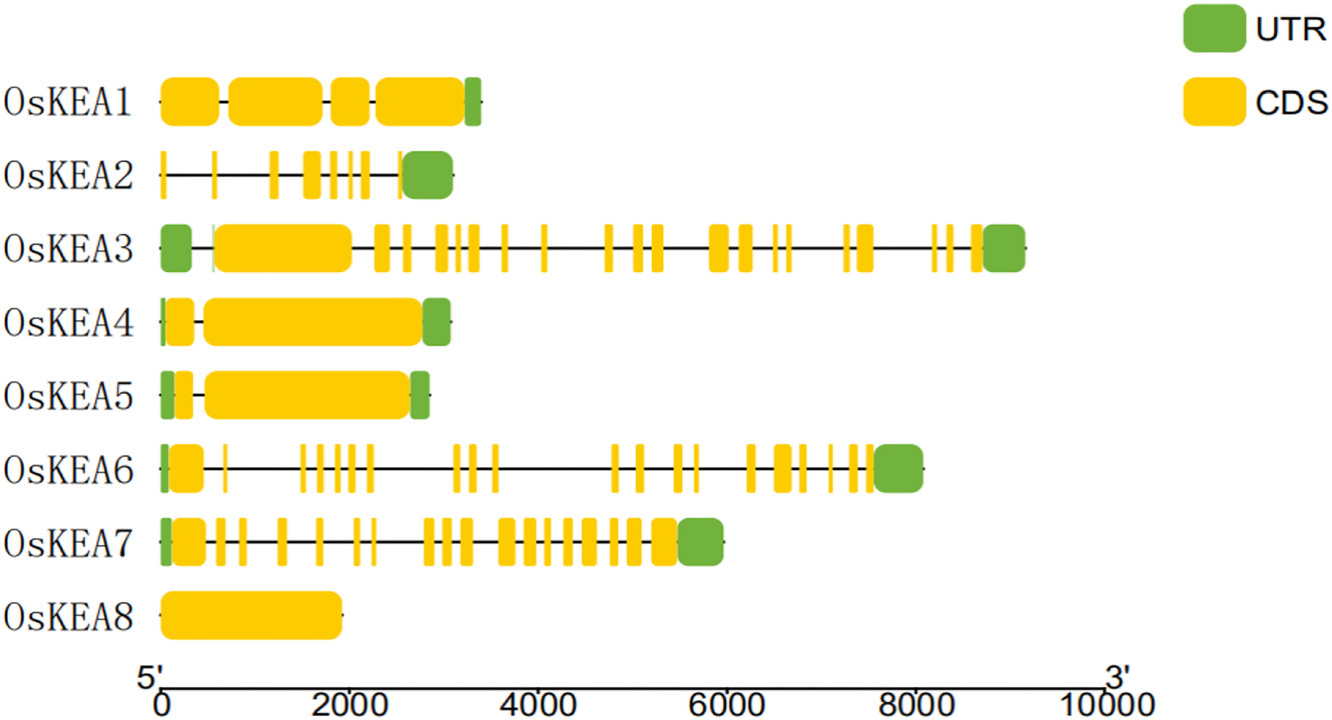
Gene structure of rice KEA gene family.

Figure 4 illustrates that the number of CDS in the KEA gene family in rice varies from a minimum of 1 to a maximum of 20. Among these, OsKEA8 lacks both the 5′ UTR and 3′ UTR regions, whereas OsKEA1 and OsKEA2 are missing only the 5′ UTR region. The remaining four genes in the KEA gene family possess both UTR regions.

**Figure 5:**
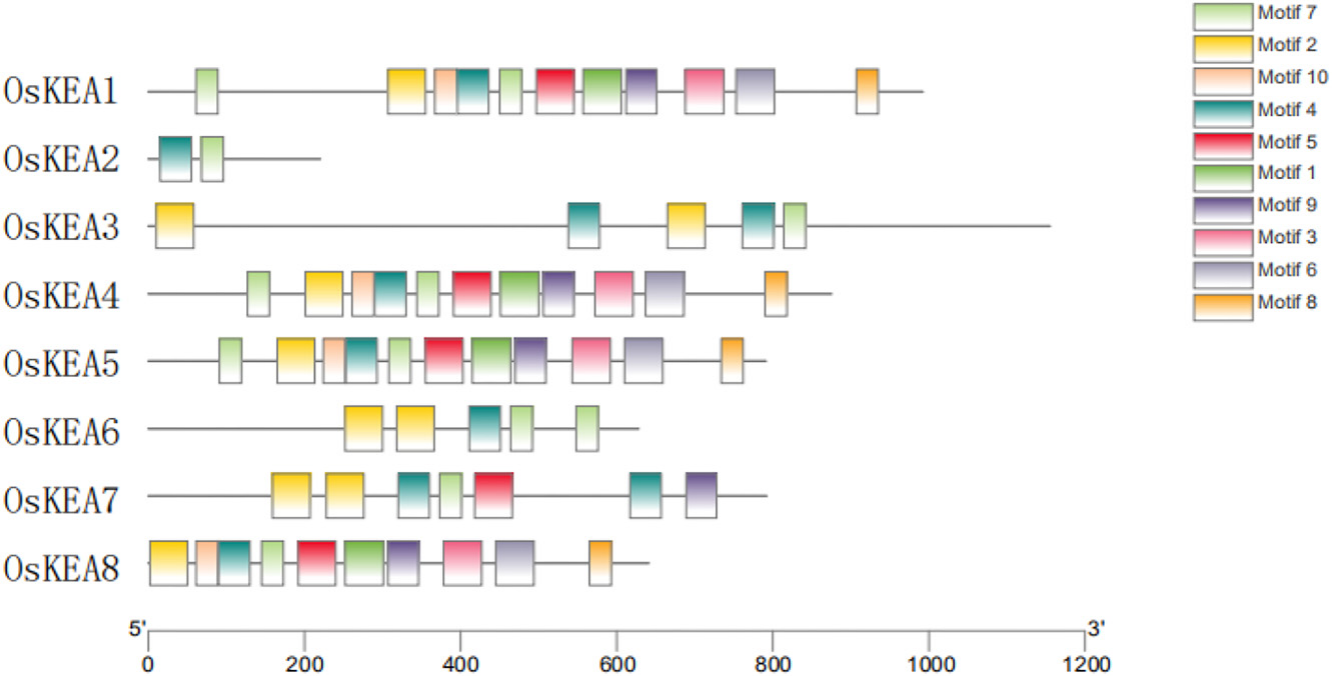
Motif of KEA gene family in rice.

As shown in Figure 5, the motifs of OsKEA1, OsKEA4, and OsKEA5 are highly similar and all include motif1, motif2, motif3, motif4, motif5, motif6, motif7, motif8, motif9, and motif10, with the highest number of motifs observed among the analyzed genes. Among these motifs, motifs 4 and 7 are commonly present across the KEA gene family.

### 3.8 Expression analysis of rice KEA genes

**Figure 6:**
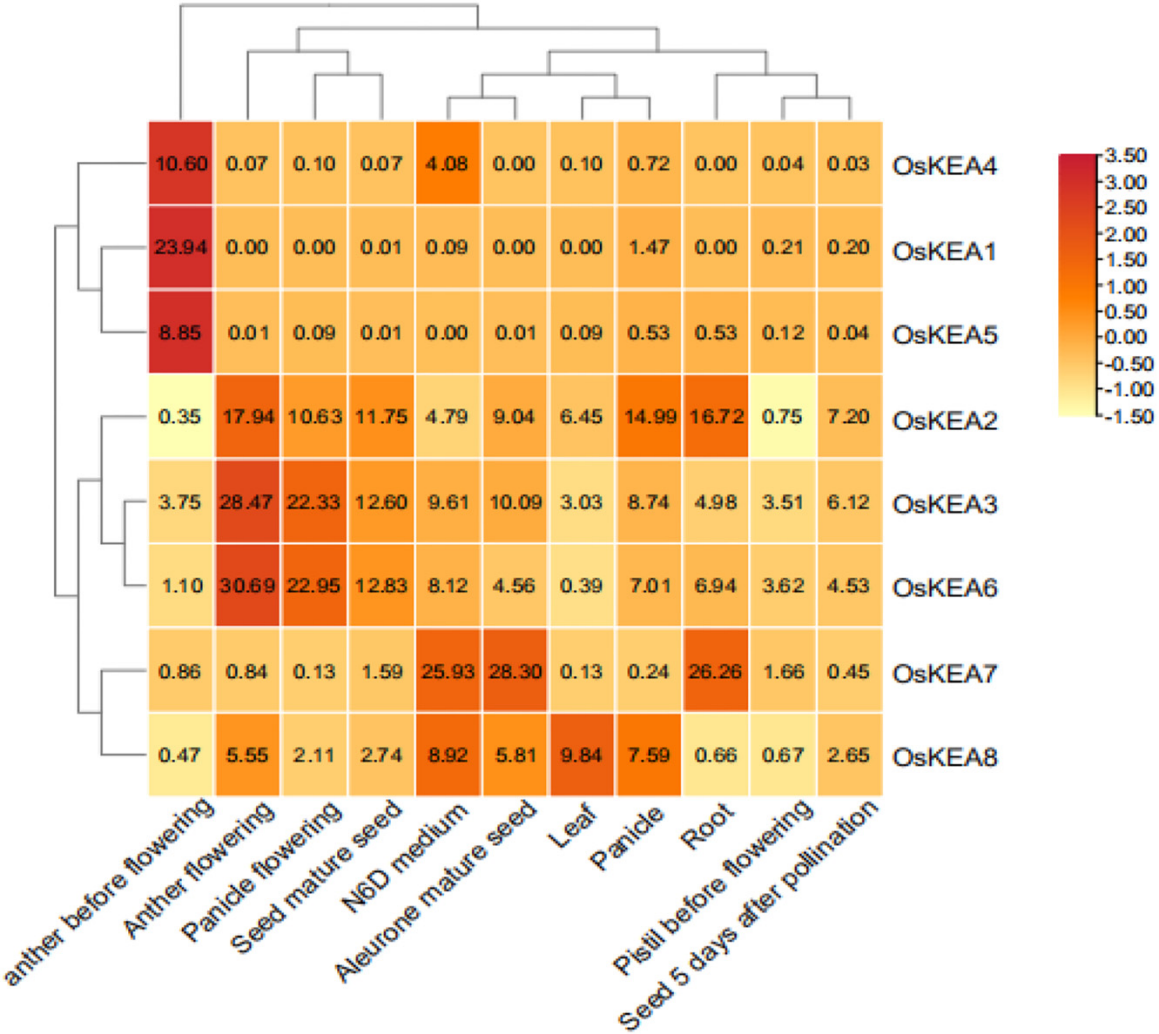
Expression profile of rice KEA gene in different tissues.

Visualize the FPKM values of the obtained genes using Rice Expression Database. Use TBtools to create a heatmap based on the FPKM values of each gene (Figure 6). The results showed that genes OsKEA1, OsKEA4, and OsKEA5 were highly expressed in the anthers before flowering, genes OsKEA2, OsKEA3, and OsKEA6 were highly expressed in the anthers and inflorescences during flowering, and gene OsKEA3 was highly expressed in all 11 tissues; For the OsKEA gene with low expression in the figure, we speculate that it may not be involved in regulating the growth and development of rice, but may participate in rice’s resistance to adverse external environments. Overall, OsKEA plays an important role in the growth and development of rice.

## 4 Discussion

K^+^ transporter genes mainly exist in the form of families in plants. In evolution, they replicate to form genes with similar functions and structures, and all transporter genes originate from the same ancestor. The KEA gene family proteins are an important transporter protein family in plants, playing a crucial role in maintaining ion balance and growth and development processes within plant cells. Currently, functional studies of the KEA gene family in plants have primarily focused on *A. thaliana*. Research has shown that when AtKEA proteins from *Arabidopsis* are individually expressed in yeast mutant strains deficient in ion transporters, AtKEA1–6 can differentially restore tolerance to high K^+^ concentrations, indicating that the AtKEA family in *Arabidopsis* possesses K^+^ transport activity [5]. Studies utilizing the *E. coli* expression system to analyze the ion transport activity of AtKEAs revealed that AtKEA1-3 and AtKEA5 exhibit bidirectional K^+^ transport activity, whereas AtKEA4 and AtKEA6 function as K^+^ uptake carriers [27]. According to GUS staining and RT-qPCR results, AtKEAs are widely expressed during plant growth and development, showing similar expression patterns. Furthermore, their expression can be induced by various environmental stresses, such as K^+^ deficiency, salt stress, and osmotic stress [5], 28], 29].

Although the KEA gene family has been studied in model plants, its members remain poorly characterized in major crops. To date, genome-wide analyses have identified 12, 24, and 6 KEA genes in soybean, wheat, and maize, respectively. In this study, bioinformatics methods were used to identify eight members of the KEA gene family in rice, and the eight family members were named OsKEA1-OsKEA8 based on their homology with AtKEA in *Arabidopsis*. The members of the gene family (6) are generally similar, indicating that the number of members of the same gene family is the same or similar among different crops of the same family and genus, suggesting diversity and differences in gene function.

According to the analysis of physical and chemical properties, the number of basic amino acids and acidic amino acids in the eight rice KEA genes is the same, both being 4, and the average hydrophobicity coefficient values are all positive. This indicates that only hydrophobic proteins are present in the members of the rice KEA gene family. The analysis of the physical and chemical properties of KEA gene family proteins shows that this gene has a high fat coefficient, indicating that the stability of rice KEA gene family proteins is relatively strong. The phylogenetic tree indicates that OsKEAs and ATKEAs members are divided into two subgroups, and the rice genes in Group II are closely related to *Arabidopsis* genes.

The research reports that have been mastered generally describe that the KEA gene has the biological function of maintaining the balance of pH and K^+^, ensuring the normal life activities of plants by maintaining their ion homeostasis. This study found through subcellular prediction of the gene family that the family proteins of this gene exist in three organelles: the cell membrane, vacuoles, and nucleus. Among them, OsKEA2, OsKEA4, OsKEA5, and OsKEA6 exist in both the cell membrane and vacuoles, indicating that this gene has specificity for different substrates. In *A. thaliana*, KEA1 and KEA2 are localized to the inner envelope membrane of chloroplasts, while KEA3 is located on the thylakoid membrane. KEA4, KEA5, and KEA6 are found in the Golgi apparatus, prevacuolar compartments, and multivesicular bodies [30]. KEA1, KEA2, and KEA3 function as chloroplast transporters involved in chloroplast development and function.

The expression patterns of rice KEA genes are highly distinctive. Notably, OsKEA1, OsKEA4, and OsKEA5 were highly expressed in the anthers before flowering, but their expression decreased significantly after flowering. In contrast, OsKEA2, OsKEA3, and OsKEA6 exhibited a completely opposite pattern: they were barely expressed in pre-flowering anthers but were highly expressed in the anthers and inflorescences during flowering. This “shift-like” expression pattern between the two groups suggests a functional division of labor during different stages of anther development. Phylogenetic analysis also revealed that OsKEA1, OsKEA4, and OsKEA5 cluster within the same clade, while OsKEA2, OsKEA3, and OsKEA6 form another distinct clade. We observed that each group shares high protein sequence similarity and exhibit analogous expression patterns. Based on these findings, we propose that these two sets of proteins may coordinately regulate anther development before and after flowering and are likely to exhibit functional redundancy. However, the above conclusion is currently solely based on bioinformatics prediction and correlation analysis, lacking the support of direct experimental evidence. Subsequent research should verify the specific functions of KEA genes through molecular genetics experiments, including gene editing, overexpression, and subcellular localization, to clarify their mechanism of action in the growth and development of rice.

The KEA gene belongs to the CPA2 subfamily of cation/proton antiporters (CPAs) and plays an important role in maintaining ion balance. This study used bioinformatics methods to systematically identify and analyze the entire rice genome and obtained a total of 8 OsKEA family genes. Exploration and analysis were conducted on its gene structure, physicochemical properties, chromosome localization, phylogenetics, and tissue-specific expression. The analysis showed that the KEA gene in rice is distributed on six different chromosomes; This family of proteins mainly exists in the cell membrane, vacuoles, and nucleus. Its expression patterns also vary in different tissues, with most genes being highly expressed in anthers before or during flowering. Although the expression profile analysis in this study revealed that multiple OsKEA genes were significantly upregulated during reproductive development, suggesting their potential involvement in this process, the specific *in vivo* functions of these genes remain unclear. The current research findings are merely preliminary speculations, and further experiments are urgently required to establish the causal relationship between KEA genes and reproductive development.

This study represents the first genome-wide identification and comprehensive analysis of the KEA gene family in rice, establishing a valuable foundation for future functional studies of these genes. The findings suggest that OsKEA genes have promising potential for rice breeding, particularly through roles in ion transport and anther development, which may contribute to improved reproductive growth and yield stability under environmental constraints.
